# Supplementary material for: Does the type of foam roller influence the recovery rate, thermal response and DOMS prevention?
Source: PLoS One. 2020 Jun 26;15(6):e0235195. doi: 10.1371/journal.pone.0235195 (PMC7319325; doi:10.1371/journal.pone.0235195)
Supplement: Attachment — Submitted filename: PONE-D-20-05356.pdf [file pone.0235195.s001.pdf]

General

The current manuscript looks to determine if there is a difference between foam roller type versus passive recovery following a bout of maximum effort jump squats. Thermal imaging was used to measure changes in blood flow via skin temperature and blood lactate was measured to gauge the effectiveness of the exercise and determine difference in immediate recovery. It was determined that the type of foam roller doesn't affect the clearance of lactate or symptoms associated with DOMS, however, it was found to show some improvements versus passive recovery. While the manuscript is well written, I do believe it needs revisions before it can be published.

COMMENTS

Abstract

Page 2, Lines 27-28: The first sentence should start out "The study…"

Page 2, Line 29: Males should be plural.

Page 2, Line 32: you should have [] around Tsk and LA to indicate abbreviations.

Introduction

General

You switch back and forth between using the term roller vs. foam roller. You should pick one and make it consistent throughout.

Page 2, Line 50: It's the first time you use the abbreviation of LA in the main body, so you need to write it out the first time, followed by the abbreviation.

Page 3, Line 52: You use the provider "Physiotherapist". However, many other healthcare professionals use foam rollers for treating athletes, so I would suggest making it more encompassing of all professions.

Page 3, Lines 63-64: Your 1st sentence is confusing. What is it compared too? Not sure this sentence should be your lead. Your paragraph is talking about arterial flow, so maybe swap the 1st and 2nd sentences.

Page 3, Line 67: Remove the 'a' from the sentence.

Page 3, Line 70: You need to write out DOMS the first time you use it. Don't assume everyone knows what DOMS stands for.

Page 4, Lines 105-107: I would include a statement about the foam rolling being a single bout so not to confuse since measurements are happening up to 96 hours.

Methods

Page 4, Line 127-128: The first sentence needs a reference.

Page 6, Line 134: You can delete IAT since you never use the abbreviation again.

Page 6, Line 142: Same as above. ART is only used here.

Page 6, Line 142: I believe you have a typo stating 20 minutes. Otherwise this sentence is very confusing.

Page 6, Lines 148-151: On your ROI you define thigh, both anterior and posterior, as going down to the ankle. Thigh is a Latin term meaning femur, so you need to change your terminology. Plus your two tight measurements include the two calf measures.

Results
General
The figures in your results should show were any significant differences occur. They should stand-alone and a reader should be able to look at just the figures and know what the results say.

How was VAS measured? Where participants asked to think of a certain area, was it general body pain? You need to include more details about the measurement of VAS. Also, do you have a VAS score prior to exercise? If assume individuals would report 0 on a VAS scale before exercise, but this is not true. Previous studies have found that participants can report anywhere from a 1-3 on the VAS scale at baseline.

Page 8, Line 209: Pain was significantly reduced in all groups when?

Discussion
Page 11, Lines 286-287: Your sentence starting off "Switching off by pressing…" is confusing. Consider revising for clarity.

You don't discuss any limitations in your discussion. I'm sure your study had some and they should always be included.

Tables
Table 2 needs clarity in the legend. IAT, ART, and AFTER30 need to be defined as figures and tables should always stand alone.
Also, you have no symbol for p<0.001 in the legend.
