# Supplementary material for: Does the type of foam roller influence the recovery rate, thermal response and DOMS prevention?
Source: PLoS One. 2020 Jun 26;15(6):e0235195. doi: 10.1371/journal.pone.0235195 (PMC7319325; doi:10.1371/journal.pone.0235195)
Supplement: Attachment — Submitted filename: plusone reveiw.docx [file pone.0235195.s002.docx]

Review for Plus one

Abstract:

The abstract is written with many format/ writing issues: 1. The first paragraph is not a paragraph, and should be developed more that just one sentence. 2. Same for paragraph 2. 3. The overall writing is very confusing and at times does flow with any sense of organization. I suggest using something like intro, methods (Assessment, Timing and Treatments) then Results and Conclusion.

Introduction:

Once again the first paragraph is not developed, and only has 2 sentences. Also the first paragraph leads me to think the paper is about lactate and Fatigue with no mention or hint of foam rolling. Paragraph 2 is also underdeveloped and needs to be expanded upon so that it discusses or sets up the paper for the topic of Foam rolling and DOMS.

The use of IT in the paper specifically the intro is very confusing. I have a hard time following what “it” is in the many sentences.

Line 73 please explain further with a reference or 2 to back of this claim.,

Reduce the use of phrases like on the other hand in the intro

Line 84, consider reorganizing to move the paragraph closer to the front of the intro.

Finally please proof read the content as there are many writing and grammar mistakes in the introduction.

Methods:

The methods section needs some rewriting as to develop more paragraphs that are not 1-2 sentences.

I am not sure about the 1 minute squat jumps, could you explain what reference or reasoning suggested this time and exercise?

Line 124: can you explain what you mean by adaption, is this meant to be a familiarization or some kind of temperature balance.

IS TSK the same as thermal imaging? They seem to be used interchangable at times. Also the same occurs with rolling, foam rolling or self myofascial release. Please clarify all these

I think it might be helpful in the writing to have less acronyms at times I had to keep going back to look at some of them that should be spelled out. ROI region of interest, or immediately after the test.

Results:

Again I suggest removing the pronoun it and make the sentences stronger by stating what it is.

There are several 1 sentence paragraphs that need to be developed or included in other sections of th results.

Discussion:

Blood lactate changes: would like to see a few more comparisons of post research on lacate to the found results. And what significance does the reference on cardio bring to this paper?

Please expand upon this section with more research refences to support or refute your findings. The massage paragraph needs to tie more into the relationship to foam rolling

Line 254 when you say the results of research are you referring to your study or the literature. It is not clear.

Overall in the temperature section, I think you need to bring in your results as the compare contrast to the references you are using in this sections

Line 286: the sentence starting with switching is not a sentence please correct

DOMS conclusion section: too many times “in light of “ is used also please expand your results to comparisons of the literature

Overall I think the concept is interesting and I think the idea of trying to determine if foam rolling (specifically a type of roller) has an effect on preventing DOMS and improving recovery could be helpful to rehabilitation and sports performance. The methods performed were adequate, however the paper is not written in way that helps to clearly set up the research question. The introduction is littered with bad grammar and improper usage of terms like it and acronyms. The discussion is not a good comparison of the your results to the current literature and creates confusion as to what is out there versus what you found.

I highly suggest some editing of this paper and or proof reading by someone else to catch the grammatical errors and typos. This would take a good project, which I liked reading about, more clear to the reader.
