# Supplementary material for: Does the type of foam roller influence the recovery rate, thermal response and DOMS prevention?
Source: PLoS One. 2020 Jun 26;15(6):e0235195. doi: 10.1371/journal.pone.0235195 (PMC7319325; doi:10.1371/journal.pone.0235195)
Supplement: Attachment — Submitted filename: Response to Reviewers.docx [file pone.0235195.s003.docx]

Warsaw, May, 1^st^, 2020

**Dear Editorial Board,**

I attached a revised version of our manuscript. The revisions have been made based on suggested changes and comments from the Reviewers. Below are our responses to each remark of the Reviewers. In the attached revised document the changes are marked on red.

**REVIEWER #1**

**General**

The current manuscript looks to determine if there is a difference between foam roller type versus passive recovery following a bout of maximum effort jump squats. Thermal imaging was used to measure changes in blood flow via skin temperature and blood lactate was measured to gauge the effectiveness of the exercise and determine difference in immediate recovery. It was determined that the type of foam roller doesn’t affect the clearance of lactate or symptoms associated with DOMS, however, it was found to show some improvements versus passive recovery. While the manuscript is well written, I do believe it needs revisions before it can be published.

**COMMENTS**

**Abstract**

Page 2, Lines 27-28: The first sentence should start out “The study…”

Page 2, Line 29: Males should be plural.

Page 2, Line 32: you should have [] around Tsk and LA to indicate abbreviations.

AUTHORS' response: Included in the text.

**Introduction**

**General**

You switch back and forth between using the term roller vs. foam roller. You should pick one and make it consistent throughout.

AUTHORS' response: This has been adressed – now we’re using foam roller throughout all manuscript.

Page 2, Line 50: It’s the first time you use the abbreviation of LA in the main body, so you need to write it out the first time, followed by the abbreviation.

AUTHORS' response: Included in the text.

Page 3, Line 52: You use the provider “Physiotherapist”. However, many other healthcare professionals use foam rollers for treating athletes, so I would suggest making it more encompassing of all professions.

AUTHORS' response: This part has been removed in order not to limit potential users.

Page 3, Lines 63-64: Your 1st sentence is confusing. What is it compared too? Not sure this sentence should be your lead. Your paragraph is talking about arterial flow, so maybe swap

AUTHORS' response: This part has been rebuilded with suggested swaping between the 1st and 2nd sentences.

Page 3, Line 67: Remove the ‘a’ from the sentence.

AUTHORS' response: Included in the text.

Page 3, Line 70: You need to write out DOMS the first time you use it. Don’t assume everyone knows what DOMS stands for.

AUTHORS' response: Included in the text.

Page 4, Lines 105-107: I would include a statement about the foam rolling being a single bout so not to confuse since measurements are happening up to 96 hours.

AUTHORS' response: Included in the text. Page 5, lines 117-118.

**Methods**

Page 4, Line 127-128: The first sentence needs a reference.

AUTHORS' response: Included in the text.

Page 6, Line 134: You can delete IAT since you never use the abbreviation again.

AUTHORS' response: It is used again in Results section both in the text and Tab. 2.

Page 6, Line 142: Same as above. ART is only used here.

AUTHORS' response: It is used again in Tab. 2.

Page 6, Line 142: I believe you have a typo stating 20 minutes. Otherwise this sentence is very confusing.

AUTHORS' response: This sentence has been improved.

Page 6, Lines 148-151: On your ROI you define thigh, both anterior and posterior, as going down to the ankle. Thigh is a Latin term meaning femur, so you need to change your terminology. Plus your two tight measurements include the two calf measures.

AUTHORS' response: I'm not sure if I understand correctly your comment on femur, as thigh refers to „upper leg”. Not the femur exclusively. Of course it’s our fault with ankle and it should be one description for thigh and second for calf. This has been clarified. Page 7, lines 161-166.

**Results**

**General**

The figures in your results should show were any significant differences occur. They should stand-alone and a reader should be able to look at just the figures and know what the results say.

AUTHORS' response: Unfortunatelly there were no significant differences in LA level (Fig. 1). On fig. 2 they are marked as follow:

**significant difference**

**STH vs PAS**

***(p=0.008)***

***------------------------***

**GRID vs PAS**

***(p=0.003)***

How was VAS measured? Where participants asked to think of a certain area, was it general body pain? You need to include more details about the measurement of VAS. Also, do you have a VAS score prior to exercise? If assume individuals would report 0 on a VAS scale before exercise, but this is not true. Previous studies have found that participants can report anywhere from a 1-3 on the VAS scale at baseline.

AUTHORS' response: This issue has been adressed in methods section. Page 7, lines 158-160.

Page 8, Line 209: Pain was significantly reduced in all groups when?

AUTHORS' response: This has been clarified in the text.

**Discussion**

Page 11, Lines 286-287: Your sentence starting off “Switching off by pressing…” is confusing. Consider revising for clarity.

AUTHORS' response: Included in the text.

You don’t discuss any limitations in your discussion. I’m sure your study had some and they should always be included.

AUTHORS' response: Included in the text.

**Tables**

Table 2 needs clarity in the legend. IAT, ART, and AFTER30 need to be defined as figures and tables should always stand alone.

AUTHORS' response: Included in the text. Legend has been clarified.

Also, you have no symbol for p<0.001 in the legend.

AUTHORS' response: Indeed, we added that.

**REVIEWER #2**

Review for Plus one

Abstract:

The abstract is written with many format/ writing issues: 1. The first paragraph is not a paragraph, and should be developed more that just one sentence. 2. Same for paragraph 2. 3. The overall writing is very confusing and at times does flow with any sense of organization. I suggest using something like intro, methods (Assessment, Timing and Treatments) then Results and Conclusion.

AUTHORS' response: Included in the text.

Introduction:

Once again the first paragraph is not developed, and only has 2 sentences. Also the first paragraph leads me to think the paper is about lactate and Fatigue with no mention or hint of foam rolling. Paragraph 2 is also underdeveloped and needs to be expanded upon so that it discusses or sets up the paper for the topic of Foam rolling and DOMS.

AUTHORS' response: Included in the text.

The use of IT in the paper specifically the intro is very confusing. I have a hard time following what “it” is in the many sentences.

AUTHORS' response: This has been included in the text as well as English grammar check has been provided by AJE. Please see reference # BFF0-200A-BB08-7F0E-1A7C

Line 73 please explain further with a reference or 2 to back of this claim.,

AUTHORS' response: Included in the text.

Reduce the use of phrases like on the other hand in the intro

AUTHORS' response: Included in the text.

Line 84, consider reorganizing to move the paragraph closer to the front of the intro.

AUTHORS' response: As above we are writing about general effects of FR, so placing this here as more detailed seems to be justified.

Finally please proof read the content as there are many writing and grammar mistakes in the introduction.

AUTHORS' response: Included in the text. Manuscript was edited for proper English language, grammar, punctuation, spelling, and overall style by AJE, reference # BFF0-200A-BB08-7F0E-1A7C

Methods:

The methods section needs some rewriting as to develop more paragraphs that are not 1-2 sentences.

AUTHORS' response: Included in the text.

I am not sure about the 1 minute squat jumps, could you explain what reference or reasoning suggested this time and exercise?

AUTHORS' response: During maximal efforts, the anaerobic (lactic) system lasts from 45 seconds to 2 minutes [[Swanwick & Matthews, 2018](https://medcraveonline.com/MOJSM/energy-systems-a-new-look-at-aerobic-metabolism-in-stressful-exercise.html#ref9)]. That is why we choose 1 min. As we could observe in our previous studies [[Adamczyk et al., 2014](https://pdfs.semanticscholar.org/56cc/ac88b6a69107ced0d9cd41444835770a826c.pdf); [Adamczyk et al., 2016](https://www.ncbi.nlm.nih.gov/pubmed/27503712)] that this time along with maximal engagement of participants creates conditions close to volitional exhausted. Along with high LA concentration (mean for all participants 10.16 ±2.87) ranged 4.8-18.9 mmol/L warrant us that this exercise will be eligible for research aim i.e. “…inducing a significant increase in blood lactate levels as an effect glycolytic nature of exercise”.

Line 124: can you explain what you mean by adaption, is this meant to be a familiarization or some kind of temperature balance.

AUTHORS' response: Following explanation was added to the manuscript:

The purpose of the adaptation was to achieve a state of thermal balance in relation to the ambient temperature so that the obtained thermograms were reliable. Thanks to this, changes in the thermal image were the result of disturbances in production or heat dissipation as a result of the exercises [[Ring & Ammer, 2012](https://www.ncbi.nlm.nih.gov/pubmed/22370242)].

IS TSK the same as thermal imaging? They seem to be used interchangable at times.

AUTHORS' response: T_sk_ means skin temperature as a result of thermal image measurement. Appropriate explanation has been included in the text.

Also the same occurs with rolling, foam rolling or self myofascial release. Please clarify all these.

AUTHORS' response: We used foam roller as a self-myofascial release technique and only in this context it has been used in the text.

I think it might be helpful in the writing to have less acronyms at times I had to keep going back to look at some of them that should be spelled out. ROI region of interest, or immediately after the test.

AUTHORS' response: To clarify this, we explained with every first use in each part of the manuscript.

Results:

Again I suggest removing the pronoun it and make the sentences stronger by stating what it is.

There are several 1 sentence paragraphs that need to be developed or included in other sections of the results.

AUTHORS' response: Included in the text.

Discussion:

Blood lactate changes: would like to see a few more comparisons of post research on lacate to the found results. And what significance does the reference on cardio bring to this paper?

AUTHORS' response: Included in the text. Page 10, lines 253-255

Please expand upon this section with more research refences to support or refute your findings. The massage paragraph needs to tie more into the relationship to foam rolling

AUTHORS' response: Included in the text. Page 10, lines 259-263 / Page 11, lines 265-267 / Page 11, lines 273-274.

Line 254 when you say the results of research are you referring to your study or the literature. It is not clear.

AUTHORS' response: Reference included in the text. Page 11, line 277

Overall in the temperature section, I think you need to bring in your results as the compare contrast to the references you are using in this sections

Line 286: the sentence starting with switching is not a sentence please correct

AUTHORS' response: Included in the text.

DOMS conclusion section: too many times “in light of “ is used also please expand your results to comparisons of the literature

AUTHORS' response: Included in the text.

Overall I think the concept is interesting and I think the idea of trying to determine if foam rolling (specifically a type of roller) has an effect on preventing DOMS and improving recovery could be helpful to rehabilitation and sports performance. The methods performed were adequate, however the paper is not written in way that helps to clearly set up the research question. The introduction is littered with bad grammar and improper usage of terms like it and acronyms. The discussion is not a good comparison of the your results to the current literature and creates confusion as to what is out there versus what you found.

I highly suggest some editing of this paper and or proof reading by someone else to catch the grammatical errors and typos. This would take a good project, which I liked reading about, more clear to the reader.

AUTHORS' response: Included in the text. Manuscript was edited for proper English language, grammar, punctuation, spelling, and overall style by AJE, reference # BFF0-200A-BB08-7F0E-1A7C
