# Supplementary material for: Does the type of foam roller influence the recovery rate, thermal response and DOMS prevention?
Source: PLoS One. 2020 Jun 26;15(6):e0235195. doi: 10.1371/journal.pone.0235195 (PMC7319325; doi:10.1371/journal.pone.0235195)
Supplement: Attachment — Submitted filename: plus one reveiw 2.docx [file pone.0235195.s004.docx]

Plus one review

Much improvement on the writing and grammar. This was much easier to ready, thank you for the rewrites and the work put into editing.

Few minor notes:

Abstract paragraph 1: add foam into the correct spots

Still a few times that “it” is used and should be edited.

Line 93: expend upon how or why Hodgson questions long term affects

Line 139: what is the (REST acronym for) do you mean that REST is equal to adaptation and thermal images. If so that seems to be confusing.

Reference: please make sure the proper reference formats are being used throughout the entire reference sections.
