# Supplementary material for: Does the type of foam roller influence the recovery rate, thermal response and DOMS prevention?
Source: PLoS One. 2020 Jun 26;15(6):e0235195. doi: 10.1371/journal.pone.0235195 (PMC7319325; doi:10.1371/journal.pone.0235195)
Supplement: Attachment — Submitted filename: Response to Reviewers2.docx [file pone.0235195.s005.docx]

Warsaw, June, 1^st^, 2020

**Dear Editorial Board,**

I attached a revised version of our manuscript. The revisions have been made based on suggested changes and comments from the Reviewers. Below are our responses to each remark of the Reviewers. In the attached revised document the changes are marked on red.

Reviewer #2:

Much improvement on the writing and grammar. This was much easier to ready, thank you for the rewrites and the work put into editing.

Few minor notes:

Abstract paragraph 1: add foam into the correct spots

AUTHORS' response: Included in lines 29 and 30.

Still a few times that “it” is used and should be edited.

AUTHORS' response: Included and edited in lines 70, 79, 335 and also “it” has been removed in lines 112, 249, 280.

Line 93: expend upon how or why Hodgson questions long term affects

AUTHORS' response: Included in lines 94-95.

Line 139: what is the (REST acronym for) do you mean that REST is equal to adaptation and thermal images. If so that seems to be confusing.

AUTHORS' response: This has been clarified in line 142, as REST is acronym of resting state before exercise.

Reference: please make sure the proper reference formats are being used throughout the entire reference sections.

AUTHORS' response: Included in the text.

Reviewer #3:

Minor revisions:
1- In the abstract, indicate that the trial was randomized.

AUTHORS' response: Included in line 33.
2- Move the sentences from lines 122-8 to the results section.

AUTHORS' response: We were not sure about this remark. Are we supposed to do this? As lines 122-8 are description of participants so we believe it should stay in this part of the paper not in results section. Anyway we added **“Participants”** subheading in here.

Indicate the type of summary statistics provided in parenthesis.

AUTHORS' response: Included in the text.

3- Line 182: typographical error: Large effects had and eta-squared “>” 0.40.

AUTHORS' response: Included in the text.

4- Provide a summary table of the results corresponding to the details contained in lines 190-9. Include summary statistics, the overall p-value from the repeated measures ANOVA, and the pairwise Bonferroni corrected p-values (similar to Table 2).

AUTHORS' response: Included in the text.

5- Table 1: To conform to standard practice, transpose the matrix. List the groups in columns at the top and the biometric characteristics in the rows. Additionally, indicate that the summary values are means and standard deviations or standard errors of the means.

AUTHORS' response: Included in the text.

6- Table 2: Indicate the type of summary statistic following the +/- sign.

AUTHORS' response: Included in the text.

7- Be consistent with the notation for T_sk_.

AUTHORS' response: We included that, one place without down script in line 243 has been corrected.
8- Page 21: Indicate if the confidence intervals shown on the graph were adjusted for repeated measures.

AUTHORS' response: Included in the text.

9- Recreate Figure 2 making it similar to Figure 1. Use a line plot and confidence intervals.

AUTHORS' response: Included in the text.

10- State and justify the study’s target sample size with a pre-study statistical power calculation. The power calculation should include: sample size, alpha level (indicating one or two-sided), minimal detectable difference and statistical testing method.

AUTHORS' response: Following statement has been added *„For the sample size (3 groups, each n = 11), assuming a typical significance level alpha = 0.05 (two-way test) and the standardized effect RMSSE = 0.77, the test power was 0.88”* – lines 181-182.
11- Indicate the date range subjects were enrolled in the study.

AUTHORS' response: The study was conducted in October 2019. Each group performed test separately but experimental sessions for groups, were held at the same time of the day. We’ve included this statement in the text, lines 127-128.

12- Clearly define the aims of the study.

Aims has been clarified in lines 118-119.
